# Meal-induced increases in C-reactive protein, interleukin-6 and tumour necrosis factor α are attenuated by prandial + basal insulin in patients with Type 2 diabetes

**DOI:** 10.1111/j.1464-5491.2011.03324.x

**Published:** 2011-09

**Authors:** P J Beisswenger, W V Brown, A Ceriello, N A Le, R B Goldberg, J P Cooke, D C Robbins, S Sarwat, H Yuan, C A Jones, M H Tan, for the IOOI Study Investigators

**Affiliations:** Section of Endocrinology, Diabetes and Metabolism, Dartmouth Medical School and Dartmouth-Hitchcock Medical CenterHanover, NH; * Emory Lipid Research Laboratory, Emory University and Atlanta VAMCAtlanta, GA, USA; † Institut d'Investigacions Biomediques August Pi i Sunyer (IDIBAPS) and Centro de Investigación Biomédica en Red de Diabetes y Enfermedades Metabólicas Asociadas (CIBERDEM)Barcelona, Spain; ‡ School of Medicine, Division of Endocrinology, Metabolism and Diabetes, University of MiamiMiami, FL; § School of Medicine, Stanford UniversityPalo Alto, CA; ¶ Lilly Research Laboratories, Eli Lilly and CompanyIndianapolis, IN, USA

**Keywords:** glycative stress, inflammation, oxidative stress, postprandial glucose, pro-inflammatory cytokines

## Abstract

**Aim:**

To determine if a regimen with prandial + basal insulin compared with basal insulin attenuates post-meal inflammatory and glycative biomarkers in patients with Type 2 diabetes.

**Methods:**

This test-meal sub-study in the USA is from a previously reported clinical trial comparing the effect on glycaemic control of 24 weeks of thrice-daily pre-meal insulin lispro mix 50 (50% insulin lispro, 50% insulin lispro protamine suspension) or bedtime insulin glargine, both plus metformin. In the sub-study, glucose, insulin, triglycerides, high-sensitivity C-reactive protein, tumour necrosis factor α, interleukin-6, methylglyoxal and 3-deoxyglucosone were measured during the post-meal period of a mixed-meal breakfast at the final visit. Prandial + basal (*n* = 25) and basal (*n* = 21) insulin were administered at the same times as during the previous 24 weeks.

**Results:**

Post-meal, the prandial + basal insulin group had significantly higher insulin, lower glucose and triglycerides, as well as lower high-sensitivity C-reactive protein, tumour necrosis factor α and interleukin-6, than the basal insulin group. Glucose incremental area under the concentration curve significantly correlated with high-sensitivity C-reactive protein, tumour necrosis factor α, interleukin-6, methylglyoxal and 3-deoxyglucosone incremental area under the concentration curve. Insulin incremental area under the concentration curve correlated inversely with high-sensitivity C-reactive protein and tumour necrosis factor α incremental area under the concentration curve. However, after adjusting for glucose incremental area under the concentration curve, these inverse correlations were no longer significant. Triglyceride incremental area under the concentration curve was not correlated with any biomarker incremental area under the concentration curve.

**Conclusions:**

Controlling post-meal hyperglycaemia with prandial + basal insulin in patients with Type 2 diabetes attenuates meal-induced increases in high-sensitivity C-reactive protein, interleukin-6 and tumour necrosis factor α compared with basal insulin. The rise in post-meal glucose, but not triglycerides, significantly correlated with the rise in post-meal inflammatory and glycative biomarkers.

## Introduction

Post-meal hyperglycaemia is considered a risk factor for cardiovascular disease in patients with Type 2 diabetes [1]. Post-meal increases in glucose and triglycerides can be associated with inflammation, oxidative stress, glycative stress and endothelial dysfunction [2–6]. Treatments that target post-meal hyperglycaemia are recommended [1], but few studies have investigated the impact of therapeutic interventions on the meal-induced inflammatory response in Type 2 diabetes. Decreasing post-meal hyperglycaemia with oral glucose-lowering agents such as mitiglinide and repaglinide can attenuate meal-induced increases in several inflammatory cytokines [7,8] and can decrease carotid intima-media thickening in Type 2 diabetes [8]. To the best of our knowledge, however, the effect of controlling post-meal hyperglycaemia with prandial + basal insulin on meal-induced high-sensitivity C-reactive protein (hsCRP), tumour necrosis factor α (TNF-α) and interleukin-6 in patients with Type 2 diabetes has not been reported.

Basal insulin is widely recommended for patients with Type 2 diabetes who cannot achieve glycaemic goals with oral glucose-lowering medications alone [9]. Because this regimen may fail to control post-meal hyperglycaemia, an alternate approach is to use prandial + basal insulin as a mixture of short- or rapid-acting and basal insulin in various ratios [10]. In individuals without diabetes, approximately 50% of daily insulin secretion is basal, while the remainder is prandial [11]. Therefore, a 50/50 mix of prandial + basal insulin can approximate normal insulin secretion and we recently showed that it can be used to control both pre- and post-meal hyperglycaemia in patients with Type 2 diabetes [12].

We now report a sub-study of the parent study [12], where our current goal is to simultaneously measure the changes in plasma hsCRP, TNF-α and interleukin-6 after a mixed-meal breakfast in patients with Type 2 diabetes and relate these changes to those of post-meal glucose, insulin and triglycerides. We postulated that controlling post-meal hyperglycaemia with a prandial + basal insulin regimen can attenuate meal-induced increases of these inflammatory cytokines. Because post-meal inflammation is potentially influenced by glycative stress [6], we also studied the concomitant changes in the alpha-dicarbonyls, methylglyoxal and 3-deoxyglucosone, in the post-meal period.

## Patients and methods

### Parent study

For logistical reasons, this test-meal sub-study was carried out in the cohort of patients enrolled by clinics in the USA who participated in the parent study, a multi-country, randomized, open-label, parallel-group, 24-week clinical trial conducted in seven countries, comparing the two insulin regimens on long-term efficacy and safety in patients with Type 2 diabetes. Fifty-six of the 96 patients from US clinics participated in this sub-study. The study was conducted according to the guidelines from the Declaration of Helsinki with approval from a local ethical review board. All patients gave written informed consent.

The entry criteria and results of the parent study have been previously reported [12]. Briefly, patients with Type 2 diabetes [ages 35–75 years; HbA_1_c 48–97 mmol/mol (6.5–11%)] using oral glucose-lowering medication(s) alone and/or with once- or twice-daily insulin were recruited. All patients were changed to a regimen containing insulin lispro mix 25 (25% insulin lispro, 75% insulin lispro protamine suspension; LM25; Eli Lilly and Company, Indianapolis, IN, USA) subcutaneously before breakfast and evening meal. This was to ensure that insulin-naïve patients would accept insulin therapy and that all patients were on the same insulin regimen when randomized. Metformin was taken orally twice daily and titrated to a maximally tolerated daily dose of 1000 to 2000 mg.

After 6 weeks of this regimen, patients were randomized to one of two groups: to receive insulin lispro mix 50 (50% insulin lispro, 50% insulin lispro protamine suspension; LM50; Eli Lilly and Company) subcutaneously thrice daily before meals (prandial + basal group) or insulin glargine (Lantus®; Sanofi-Aventis, Paris, France) subcutaneously once daily at bedtime (basal group). Metformin was continued unchanged in both regimens. Both insulins were adjusted to achieve a target fasting blood glucose level < 6.7 mmol/l (120 mg/dl). In addition, patients in the prandial + basal group targeted a 2-h post-meal blood glucose level < 8.0 mmol/l (144 mg/dl). The diet, exercise and concomitant medications plan was not altered during the study.

### Test-meal study

To assess the meal-induced responses of the biomarkers at baseline and ensure that the two groups had similar post-meal responses, test meal 1 was given at the end of the lead-in period (2–10 days before randomization). After continuing the specified insulin regimens for 24 weeks, test meal 2 was conducted to test the study hypothesis.

The standardized test-meal breakfast consisted of a standard commercial breakfast [one bacon, egg and cheese biscuit, one hash brown patty and 8 oz (237 ml) of orange juice], containing 39 g fat, 78 g carbohydrate, 24 g protein and 750 kCal. The night before test meal 1, patients ate their usual evening meal and fasted overnight. All patients took metformin 30 min before and injected LM25 15 min before breakfast. The night before test meal 2, patients ate their usual evening meal and fasted overnight. All patients took metformin 30 min before the test meal. Patients treated with insulin glargine injected it at bedtime the previous night and those treated with LM50 injected it 15 min before breakfast, as they had been doing for the previous 24 weeks. The lunchtime dose of LM50 was omitted on the study day. After the test-meal breakfast, patients were placed under observation and were not allowed to eat for the next 8 h, but had free access to water.

Blood samples were collected 5 min before the test meal (0 h) and at 1, 2, 3, 4, 6 and 8 h after to measure the levels and timing of post-meal responses. Levels of plasma glucose, insulin, triglycerides, chylomicron triglycerides, hsCRP, TNF-α and interleukin-6 were measured at each time point. Methylglyoxal and 3-deoxyglucosone were measured at 0, 1, 2 and 3 h as previously reported [6,14]. Specimens were shipped to a central laboratory and then to specialized laboratories for analysis as required.

HbA_1c_ was measured using high-performance liquid chromatography (Variant; Bio-Rad Laboratories, Munich, Germany) at Covance Central Laboratory (Indianapolis, IN, USA). Glucose was determined using a hexokinase method (Roche Modular Analyser; Roche, Indianapolis, IN, USA). Insulin was measured with a micro-particle enzyme immunoassay (IMX Insulin reagent pack; Abbott Laboratories, Abbott Park, IL, USA). Immunonephelometry with the hsCRP reagent from Dade Behring Inc., (Newark, DE, USA) was used to measure hsCRP. Commercially available ELISA kits (Bender MedSystems Diagnostics GmbH, Vienna, Austria) were used to assay interleukin-6 and TNF-α [13]. Methylglyoxal and 3-deoxyglucosone were assayed as previously described [6,14]. Plasma and chylomicron triglyceride levels were determined by enzymatic methods with a Beckman Synchron CX7 chemistry analyser (Beckman Coulter Inc., Brea, CA, USA). Chylomicron fractions (Sf > 400) were isolated using a two-step ultracentrifugation as previously described [15].

### Study outcomes

The outcomes were comparisons between treatment groups for the plasma glucose, insulin, triglyceride, chylomicron triglyceride, hsCRP, TNF-α, interleukin-6, methylglyoxal and 3-deoxyglucosone concentrations at each time point. Post-meal total area under the concentration curve (AUC) and incremental AUC of each biomarker during the test meal were also compared. The incremental AUC measured the integrated excursion during the post-meal period by subtracting the fasting value from each parameter value at the designated time during the sampling period.

### Statistical analysis

Based on a previous report that post-meal triglyceride change is associated with changes in interleukin-6 and TNF-α [4], the power calculation for the test-meal sub-study was based on the AUC for chylomicron triglycerides. The study required 48 patients, 24 in each treatment group, to allow detection of a 9.6% between-treatment difference in the change from baseline AUC, tested using a two-sided test at a significance level of 0.05 with 90% power.

Analyses were performed on data from those patients who completed both test meals: one before randomization and the other after they had been on their study insulin regimen for 24 weeks. Patient characteristics and demographic variables were summarized by treatment group and compared using a two-sample *t*-test for the numeric measurements and a χ^2^-square test for the categorical measurements. Statistical analyses for total plasma and chylomicron triglycerides were performed using a two-sample *t*-test if the normality test passed. The Mann–Whitney rank sum test was used if the normality test failed (SigmaStat; Systat Software Inc., Chicago, IL, USA). All other analyses were conducted using SAS version 8 (SAS Institute Inc., Cary, NC, USA). No adjustments for missing data were performed except for the last-observation-carried-forward. An analysis of covariance model was used to compare treatment differences. The test-meal fasting analysis included the corresponding fasting value at the test meal before randomization as a covariate. For the total and incremental AUC analysis, the corresponding test-meal baseline value was used as a covariate and treatment was considered as fixed effect.

The incremental AUC of the patients who had both test meals was analysed for statistically significant associations with the variables. Multiple linear regression models were applied to assess the independent effect of post-meal plasma glucose, insulin or triglycerides (the independent variables) on hsCRP, TNF-α, interleukin-6, methylglyoxal and 3-deoxyglucosone. Statistical adjustments were made for fasting plasma glucose or insulin and HbA_1c_, age, gender and BMI covariates. Partial correlation analysis was performed to explore the strength and direction of the association between plasma glucose, insulin and triglycerides with hsCRP, TNF-α, interleukin-6, methylglyoxal and 3-deoxyglucosone while adjusting for fasting plasma glucose or insulin and HbA_1c_, age, gender and BMI covariates.

## Results

### Patient baseline characteristics and disposition

Fifty-six patients completed test meal 1 at the end of the lead-in period before randomization. Ten patients discontinued the study before test meal 2 at 24 weeks: four prandial + basal patients (two because of protocol violation, one because of an adverse event and one because of patient decision) and six basal patients (two because of perceived lack of efficacy, two because of move or loss of contact, one because of protocol violation and one because of patient decision). Among those completing both test meals, 25 patients were in the prandial + basal group and 21 patients were in the basal group. Analysis included data from these 46 patients.

Baseline patient characteristics were similar in both treatment groups, except for younger age in the prandial + basal group (55.1 vs. 59.5 mean years, *P* = 0.04) (Table 1). In this sub-study, 10 (40%) of the 25 patients taking LM50 switched from LM50 to LM25 as their pre-evening meal injection. In the parent study, 53 (33.8%) of the 157 patients taking LM50 switched their pre-evening meal injection to LM25 [12]. There were no differences in baseline demographics (age, gender, ethnicity) between the 40 US patients who did not participate in this sub-study and the 46 who participated in both test meals.

**Table 1:** Baseline characteristics: patients who completed both test meals*

Characteristic	Prandial + basal†*n* = 25	Basal‡*n* = 21	*P*-value
Age, years	55.1 (7.5)	59.5 (6.9)	0.04
Male gender, *n* (%)	15 (60.0)	15 (71.4)	0.42
Ethnicity, %			0.41
White	56	52	
African descent	12	24	
East or south-east Asian	0	5	
West Asian	8	0	
Hispanic	24	19	
Weight, kg	101.3 (25.2)	95.7 (21.2)	0.43
BMI, kg/m^2^	35.1 (7.7)	32.1 (6.8)	0.18
HbA_1c_, mmol/mol	56 (16)	55 (16)	0.42
HbA_1c_, %	7.3 (0.7)	7.2 (0.7)	
Duration of disease, years	11.1 (5.6)	13.4 (7.1)	0.23
Daily insulin injections before study, %			0.96
0	36	33	
1	16	19	
2	48	48	

* Represents data collected at randomization (parent study). Data are mean (standard deviation) unless otherwise indicated.

† Insulin lispro mix 50 plus metformin group.

‡ Insulin glargine plus metformin group.

### Test meal 1 at end of lead-in period

There were no significant differences between the two groups at this test meal with respect to mean plasma concentrations for plasma glucose, insulin, triglycerides, chylomicron triglycerides, hsCRP, TNF-α, interleukin-6, methylglyoxal and 3-deoxyglucosone. There were no significant differences between groups in post-meal total AUC or incremental AUC for any of the biomarkers. Thus, both groups, while on the same insulin regimen before randomization, had similar responses to the mixed-meal breakfast.

### Test meal 2 after 24 weeks of treatment

We compared the concentration of each biomarker at each time point and its incremental AUC of the entire post-meal period between the prandial + basal and basal groups.

#### Plasma glucose and insulin

**FIGURE 1:**
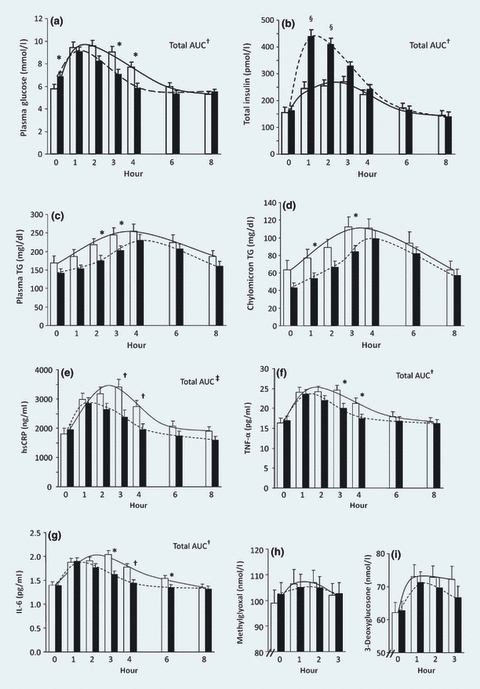
Plasma levels of glucose, insulin, TG (plasma/chylomicron), hsCRP, TNF-α, interleukin-6, methylglyoxal and 3-deoxyglucosone after test meal 2. Prior to the test meal, patients were treated for 24 weeks with insulin lispro mix 50 plus metformin (black bar with dashed trend line, *n* = 25) or insulin glargine (white bar with solid trend line, *n* = 21) plus metformin. Results are the mean ± standard error of values measured before (0 h) and after the test meal. Comparisons were between treatments at each time point and between treatments for total AUC. *P< 0.05, †*P*< 0.01, ‡*P*< 0.001, §*P*< 0.0001. AUC, area under the concentration curve; hsCRP, high-sensitivity C-reactive protein; IL-6, interleukin-6; TG, triglyceride; TNF-α, tumour necrosis factor alpha.

The prandial + basal group had significantly higher mean fasting plasma glucose, but significantly lower plasma glucose at 3 and 4 h after the meal (Fig. 1a). The prandial + basal group also had significantly lower mean post-meal plasma glucose total AUC and incremental AUC (Fig. 1a and Table 2).

**Table 2:** Comparison between the two insulin treatment groups of post-meal incremental area under the curve (AUC) of various biomarkers in test meal 2 at week 24 after randomization*

Biomarker	Prandial + basal† (*n* = 25)	Basal‡ (*n*= 21)	*P*-value
Glucose (mmol/l)·min	323.8 (77.1)	902.8 (98.6)	< 0.001
Insulin (pmol/l)·min§	46 638 (6813.1)	21 418 (5415.5)	< 0.01
TG (mg/dl)·min	23 962 (2661.8)	25 975 (3074.6)	0.46
CTG (mg/dl)·min	15 387 (1845.5)	14 414 (2141.3)	0.99
hsCRP (ng/ml)·min	75 599 (38 756.2)	377 947 (70 693.6)	< 0.001
TNF-α (pg/ml)·min	874.1 (235.8)	2150.1 (435.3)	< 0.01
IL-6 (pg/ml)·min	59.0 (21.3)	138.3 (33.8)	< 0.01
3-DG (nmol/l)·min	1014 (277.2)	1597 (260.9)	0.14
MG (nmol/l)·min	339.6 (310.4)	1004.3 (410.2)	0.23

3-DG, 3-deoxyglucosone; CTG, chylomicron triglycerides; hsCRP, high-sensitivity C-reactive protein; IL-6, interleukin-6; MG, methylglyoxal; TG, plasma triglycerides; TNF-α, tumour necrosis factor alpha.

* Data are mean (sem). The *P*-value calculation included treatment as a fixed effect and baseline value as a covariate.

† Insulin lispro mix 50 plus metformin.

‡ Insulin glargine plus metformin.

§ Insulin incremental AUC: prandial + basal group, *n* = 24; basal group, *n* = 20.

The prandial + basal group had significantly higher mean plasma insulin at 1 and 2 h and significantly higher mean post-meal plasma insulin total and incremental AUC (Fig. 1b and Table 2).

#### Plasma and chylomicron triglycerides

The prandial + basal group had significantly lower mean plasma triglyceride concentrations at 2 and 3 h after the meal (Fig. 1c) and significantly lower chylomicron triglycerides at 1 and 3 h (Fig. 1d). However, there were no significant differences between groups in post-meal total or incremental AUC for plasma or chylomicron triglyceride (Fig. 1c, d and Table 2).

#### Markers of inflammation

The prandial + basal group had significantly lower plasma hsCRP and TNF-α concentration at 3 and 4 h (Fig. 1e, f) and interleukin-6 at 3, 4 and 6 h after the meal (Fig. 1g). The prandial + basal group also had significantly lower total and incremental AUC for each of these markers (Fig. 1e, f, g and Table 2).

#### Methylglyoxal and 3-deoxyglucasone

Although they trended lower in the prandial + basal group, there were no significant differences between groups in plasma levels of these biomarkers at any time point or for total or incremental AUC after the meal (Fig. 1h, i and Table 2)

#### Regression analysis and correlation analysis for meal-induced biomarker changes (Table 3)

##### Glucose

The post-meal incremental AUC for glucose was significantly associated with post-meal incremental AUC for hsCRP, TNF-α, interleukin-6, methylglyoxal and 3-deoxyglucosone. It was not associated with the incremental AUC for insulin and triglycerides (Table 3).

**Table 3:** Regression and correlations for plasma glucose and insulin with biomarkers: incremental area under the curve (AUC)

	PG Incremental AUC			Insulin Incremental AUC		
		
Variable	Regression coefficient*	*P*-value*†	*R*†	Regression coefficient*	*P*-value*†	*R*†
Insulin‡	−8.23	0.40	−0.14	—	—	—
TG‡	−4.16	0.41	0.14	−0.01	0.85	−0.03
hsCRP‡	419.51	< 0.001	0.68	−3.71	0.01	−0.39
TNF-α‡	1.46	0.01	0.41	−0.02	0.02	−0.38
IL-6‡	0.14	< 0.01	0.46	−0.01	0.14	−0.24
3-DG¶	3.34	< 0.001	0.62	0.00	0.98	< 0.01
MG¶	2.65	0.03	0.34	−0.03	0.08	−0.28

3-DG, 3-deoxyglucosone; AUC, area under the concentration curve; hsCRP, high-sensitivity C-reactive protein; IL-6, interleukin-6; MG, methylglyoxal; PG, plasma glucose; *R*, partial correlation coefficient; TG, plasma triglycerides; TNF-α, tumour necrosis factor alpha.

* Multiple linear regression approach was applied after adjusting for covariates (fasting PG or insulin; HbA_1c_, age, gender and BMI).

† Partial correlation controlling for covariates (fasting PG or insulin; HbA_1c_, age, gender and BMI).

Incremental AUC time period:

‡ 0–8 h

¶ 0–3 h.

The *P*-value applies to both regression and correlation analysis.

##### Insulin

The post-meal incremental AUC for insulin was significantly inversely associated with the post-meal incremental AUC for hsCRP and TNF-α (Table 3). However, this relationship was no longer seen after adjusting for the post-meal glucose incremental AUC by multivariate analysis.

##### Triglycerides

The incremental AUC for triglycerides was not significantly associated with the respective post-meal incremental AUC for glucose, insulin or any of the biomarkers (data not shown).

## Discussion

The thrice-daily pre-meal prandial + basal regimen resulted in higher plasma insulin, lower plasma glucose as well as lower meal-induced increases in hsCRP, interleukin-6 and TNF-α concentrations post-meal when compared with a once-daily basal insulin regimen. There were also significant decreases of incremental AUC of inflammatory biomarkers (hsCRP, interleukin-6, TNF-α) associated with the incremental AUC of glucose. The strong correlations between attenuation of the acute rise in post-meal glucose and inflammatory markers suggest that glucose may be a factor in their release. This is the first study to show that controlling post-meal hyperglycaemia with a prandial + basal insulin regimen vs. a basal insulin regimen can attenuate the meal-induced increases in hsCRP, interleukin-6 and TNF-α in patients with Type 2 diabetes on metformin.

Previous studies on post-meal changes in inflammation in patients with Type 2 diabetes showed varied results. For example, a high-fat breakfast increased post-meal plasma interleukin-6 and TNF-α [4], whereas a mixed meal decreased interleukin-6 and TNF- α in another study [5]. A commercial meal equivalent meal increased post-meal hsCRP, but not interleukin-6 [16] and was partially confirmed by a report that a high-fat and high-glucose meal increased plasma interleukin-6 and hsCRP [7]. Two previous studies on the relationships between the meal-induced changes in inflammatory markers relative to plasma triglycerides and glucose have also shown mixed results in patients with Type 2 diabetes. Nappo *et al*. showed a significant relationship between post-meal changes in triglycerides and TNF- α/interleukin-6 and between post-meal changes in glucose and interleukin-6 [4], while Carroll and Schade found no such correlations [16].

Hyperglycaemia has previously been shown to be a major initiator of glycative (dicarbonyl) stress [6,14] and the significant relationship of methylglyoxal and 3-deoxyglucosone with post-meal glucose in this study confirms this relationship. Cause-and-effect relationships have also been shown to exist between inflammation and glycative stress [17,18], suggesting that the elevation of methylglyoxal and 3-deoxyglucosone seen in this study could initiate inflammation in the post-meal state. In the current study, however, we observed lower, but not statistically significant, methylglyoxal and 3-deoxyglucosone incremental AUC group differences in the prandial + basal group relative to the basal group. These findings may be partly attributable to substantial inter-subject data variability and the modest sample size of the study populations. Our decision, based on prior studies [6,14], to measure methylglyoxal and 3-deoxyglucosone only during the initial 3 h of the post-meal period could also have led us to miss post-meal changes at later time points, where significant changes were observed for other biomarkers.

Our finding of an inverse correlation between insulin incremental AUC and that of three inflammatory biomarkers supports the hypothesis that insulin has anti-inflammatory effects [19,20]. The loss of this effect when the insulin data were corrected for glucose effect by multivariate analysis suggests that this apparent correlation may be primarily related to glucose change. Dandona *et al*. have reported that insulin has direct anti-inflammatory effects, but, to the best of our knowledge, Dandona *et al*. [19] did not correlate the magnitude of the change of anti-inflammatory effect with the change in insulin dose or plasma insulin level. Nor did they correct for the effect of glucose in their studies on the anti-inflammatory effects of insulin. In one study of patients with acute myocardial infarction without diabetes, the rise in inflammation was attenuated in the group receiving insulin infusion compared with the group not receiving insulin, although glucose levels were similar in both groups [21]. Our study differs from these other studies in the aims and designs of studies, study subjects (subjects with Type 2 diabetes vs. subjects without diabetes), meal challenge (mixed meal vs. oral glucose [19], except for one study using mixed meal [20]) and use of constant insulin infusion vs. bolus insulin analogue.

Although we observed a modest improvement in post-meal plasma and chylomicron triglyceride concentrations with pre-meal prandial + basal over the first 3 h of the post-meal period, we did not see a relationship between changes in plasma triglyceride concentrations and changes in inflammatory markers or glycative stress. This outcome differs from the findings of Nappo *et al*. who showed that post-meal triglyceride change is related to changes in interleukin-6 and TNF-α [4]. Possible explanations for the difference between our study vs. that of Nappo *et al*. include the amount of fat consumed (39 vs. 50 g), the type of fat consumed, the duration of diabetes (11–13 years vs. newly diagnosed) and the type of correlation analysis used (area under the curve over 8 h vs. presumed individual time points). Another study, also using a standard commercial meal, reported no correlation between changes in post-meal triglycerides and those of hsCRP and IL-6 [16].

A possible limitation of this study is the age difference between the treatment groups, which could contribute to the differences in biomarker changes. This is unlikely, however, because of the relatively small difference in age between the two groups and our correction by statistical adjustment for age in the regression and correlation analyses. Other factors known to affect inflammatory markers (gender, duration of diabetes or treatment, lipid-altering and anti-hypertensive medications) were similar in both insulin treatment groups. The two groups also did not differ in weight before randomization, nor did either group show significant weight gain during the 24 weeks in this sub-study. It is unlikely that differences between the two groups in HbA_1c_ at 24 weeks influenced the acute response in inflammatory biomarkers, because both groups had good overall glycaemic control [HbA_1c_ of 46 vs. 52 mmol/mol (6.4 vs. 6.9%)]. Similarly, we do not believe that fasting blood glucose differences (8.2 vs. 6.3 mmol/l) influenced the acute response. Esposito *et al*. reported that changes in hsCRP and IL-6 correlated with changes in 2-h post-meal glucose and not with fasting plasma glucose or HbA_1c_ [8], and Festa *et al*. also reported that CRP is more strongly related to post-load oral glucose tolerance testing than to fasting glucose in subjects without diabetes [22]. In the current study, statistical adjustments were also made for fasting plasma glucose or insulin and HbA_1c_, age, gender and BMI covariates in all of the regression and correlation analyses to assure that the reported correlations were independent of these factors.

The intent of this study was to compare biomarker responses to a mixed-meal breakfast after 24 weeks of treatment with two insulin regimens and to test the hypothesis that controlling post-meal hyperglycaemia with prandial + basal insulin attenuates the meal-induced increases in hsCRP, interleukin-6, TNF-α, methylglyoxal and 3-deoxyglucosone more than basal insulin in patients with Type 2 diabetes. The test meal before randomization was performed to ensure the two groups were similar in their post-meal responses at baseline, while the test meal after 24 weeks of treatment was performed to test the study hypothesis.

In summary, our study shows that controlling post-meal hyperglycaemia with prandial + basal insulin attenuates the meal-induced increases in hsCRP, TNF-α and interleukin-6 compared with basal insulin once daily, both plus metformin. We also demonstrated that post-meal changes in hsCRP, TNF-α, interleukin-6, methylglyoxal and 3-deoxyglucosone levels are significantly correlated with post-meal glucose. As people consuming three meals daily are in the postprandial state for most of the 24 h, these findings suggest that controlling post-meal glucose can potentially have a beneficial effect on cardiovascular events in patients with Type 2 diabetes. Cardiovascular outcome studies where post-meal hyperglycaemia and meal-induced inflammation are reduced over a longer time period are needed to prove that these changes can decrease vascular disease in patients with Type 2 diabetes.

## Abbreviations

AUC: area under the concentration curve
hsCRP: high-sensitivity C-reactive protein
TNF-α: tumour necrosis factor alpha
